# The morphogenetic cross road of development and evolution: remembering Pere Alberch (1954–1998)

**DOI:** 10.1098/rsfs.2024.0033

**Published:** 2024-10-25

**Authors:** Alfonso Martinez Arias

**Affiliations:** ^1^ Systems Bioengineering, MELIS, Universidad Pompeu Fabra, Doctor Aiguader, 88, ICREA, Pag Lluis Companys 23, Barcelona, Spain

**Keywords:** morphogenetic, cross, development, evolution, Pere Alberch

Ever since the modern synthesis, evolution is more often than not seen through the lens of genes: allelic frequencies, changes in gene sequence and also relationships between specific genes and function. From the beginning, this view excluded what one could call ‘structuralist’ arguments, that were very common during the nineteenth century. From this perspective, form and embryonic development fuelled the arguments in search of principles that could relate organisms to each other. And, these arguments went a long way, creating evolutionary relationships that genes have finessed but not overturned. A relationship between development and evolution has never been disputed but the modern synthesis relegated it to background discussions. This is probably because, when you come to think of it, shape and form, patterns, are not linearly related to genes.

The notion that embryos tell us something about evolution was first hinted at by Karl Ernst von Baer in his comparative studies. His laws about the order of appearance of characters (from more general to more specific) and the scaffolding nature of structures in the evolution of body plans had an impact in biological thought. Darwin, in his great book, used similarities and divergences of embryos to support his theory of descent with modification. These ideas were later turned into a famous mantra by Ernst Haeckel when he stated, for the most part wrongly, that during development animals revisited their evolutionary history: ontogeny recapitulates phylogeny. Much later in his monumental ‘Ontogeny and phylogeny’, Gould arbitrated many disputes on the topic in favour of von Baer and interpreted Darwin in this light, leaving ideas about recapitulation by the side.

What all these views have in common is the observation that animals appear to be variations on specific structural themes. This is clearly seen in the relationship between fins, wings and limbs but it also applies to different organs that appear to progress from simple to complex, e.g. the heart, from the dual-chamber organ in fish to the complex machine with four chambers that is in mammals. The word ‘constraint’ hovers over these discussions. Is what we see that exists and has existed the only variation possible? Is the natural world we live in inevitable? The question is important to understand both animals and plants, but is more often than not discussed in animals.

It has been said that from the perspective of animals, the great crucible was the Burgess shale and that the world as we know it came out from those ponds under the close supervision of selection. It is to that place where, beholding the panoply of forms around us, we can trace back their overall segmental morphology, with their openly bilaterally polarized body plans, as well as probably their different organs, tissues and appendices. However, the mechanisms that generate novelties and evolve those novelties remain an open question. Is there a slot machine of shapes operating in the background from which the environment selects what works? Or does the environment only see a small subset of forms because not all forms are possible? We have learnt that, from the perspective of genetics, there are no constraints, that given enough time myriads of possibilities can be, and are probably being exploited. However, is this possible from the point of view of multicellular organization? Or, are there large structural constraints that limit that variation? Gould famously put it that were we to replay the tape of life, the Earth would look a very different place. Maybe. For others, maybe not.

The focus on genes has relegated these questions to background in discussions on the topic. However, they have not been forgotten. One man in particular, the Catalan biologist Pere Alberch (1954–1998), had them at the centre of his thoughts and work. He argued for the role of structural constraints in the emergence and evolution of animal form and provided examples, sometimes with experiments. Furthermore, at a time when the gene’s eye view of life was starting to permeate developmental biology in the form of evo–devo, he championed a different view, one which did not need genes but required deep thoughts about morphogenesis. In November 2023, a symposium was held at the PRBB in Barcelona, to celebrate the 25th anniversary of Alberch’s untimely death. The speakers, a mixture of students, collaborators and followers, explored his ideas and legacy through the memories of colleagues and the new findings and ideas of young scientists. The meeting made clear that Alberch was prescient as much of his research plan is being implemented now through new approaches to the questions posed by morphogenesis.

This issue of *Interface Focus* contains three articles that are representative of the symposium, Alberch’s ideas and his legacy. He made clear his position as an internalist, someone who believed that development was highly controlled by internal morphogenetic programmes that created large constraints for the evolution of form and function. His views did not dwell on genes and did not see genes as the main drivers of evolutionary morphological shape. Instead, he looked at the architecture of the organism and tried to extract the rules that run the emergence of shape and form. He developed ideas and exploited the notion of morphogenetic spaces. In his classic ‘The logic of monsters’ [1], he explores these ideas to the full. The symposium in Barcelona explored many of them, going beyond evo–devo, more concerned with creating a catalogue of genes and function across organisms, and moving into the realm of the properties and possibilities of cell ensembles.

The three articles here cover well the past, the present and the future of Alberch’s ideas. Laura Nuño de la Rosa, who has worked with close collaborators of Alberch, reviews the origin of his ideas and reminds us of his legacy. For his part, Ricard Sole and colleagues undertake a tour de force in exploring the idea of constraints at different scales, from the poorly thermodynamics to the ecological, searching to challenge whether there are other worlds around. Finally, Dominica Cao *et al*. take the logic of monsters into the twenty-first century and use stem cell embryo models to explore the internal constraints of developmental systems. Morphogenesis so much at the centre of developmental biology now was the centre and attractor of Alberch’s ideas.

In the end, it might be that there is a big challenge in trying to bring together development and evolution for, as Denis Duboule has put it, they each deal with a very different process: development is recursive, evolution is linear and open-ended [2]. This difference creates a difficulty when trying to build a bridge between the two. However, it might all be a matter of time scales. Development is recursive only in the short term. In geological time frames, mutation and selection do their job, development changes and organisms evolve. The question is the one that, probably, was in Alberch’s head, namely do the obvious structural constraints steer the workings of selection? To progress towards the answer, Alberch might have suggested, might not be to look at genes, even if at the end we will have to bring them into the picture.

## Data Availability

This article has no additional data.
